# *FLO* Genes Family and Transcription Factor *MIG1* Regulate *Saccharomyces cerevisiae* Biofilm Formation During Immobilized Fermentation

**DOI:** 10.3389/fmicb.2018.01860

**Published:** 2018-08-23

**Authors:** Leyun Yang, Cheng Zheng, Yong Chen, Hanjie Ying

**Affiliations:** ^1^State Key Laboratory of Materials-Oriented Chemical Engineering, College of Biotechnology and Pharmaceutical Engineering, Nanjing Tech University, Nanjing, China; ^2^National Engineering Research Center for Biotechnology, College of Biotechnology and Pharmaceutical Engineering, Nanjing Tech University, Nanjing, China

**Keywords:** *Saccharomyces cerevisiae*, transcription factor *MIG1*, flocculation genes, biofilm, ethanol resistance

## Abstract

*Saccharomyces cerevisiae* immobilization is commonly used for efficient ethanol fuel production in industry due to the relatively higher ethanol stress resistance of *S. cerevisiae* in biofilms relative to planktonic cells. The mechanisms of biofilm formation and stress resistance, however, remain ambiguous. By analyzing biofilm and planktonic cell transcriptomes, this study observed that *MIG1* (encoding a transcription factor) expression in cells increases during the biofilm formation process. To identify the role of *MIG1* in yeast biofilm formation and the ethanol resistance of these cells, *MIG1* was deleted and complemented in *S. cerevisiae* 1308. Results showed the *MIG1* deletion mutant strain demonstrated weaker biofilm formation ability both on fibers and plastic than the wild-type and these could be restored by expressing *MIG1* in deletion mutant. To verify the ability of *MIG1* to regulate the expression of *FLO* genes, which encode adhesions responsible for yeast biofilm formation, *FLO* gene transcription levels were measured via qRT-PCR. Relative to wild-type *S. cerevisiae*, the adhesion genes *FLO1, 5*, and *9* which also demonstrate increased expression in the transcriptome of yeast cells during biofilm formation, but not *FLO11*, were down-regulated in the *MIG1* mutant strain. Additionally, the *MIG1* mutant lost a majority of its flocculation ability, which depended on cell-cell adhesions and its slightly invasive growth ability, dependent on cell-substrate adhesion. Deleting *FLO1, 5*, and *9* decreased biofilm formation on plastics, suggesting these *FLO* genes contribute to the biofilm formation process alongside *FLO11*. Moreover, the ethanol tolerance of yeast decreased in the *MIG1* deletion mutant as well as the *FLO11* deletion mutant, resulting in reduced biofilm formation during fermentation. It remains possible that in the later period of fermentation, when ethanol has accumulated, an over-expression of the *FLO1, 5*, and *9* genes regulated by *MIG1* would enhanced cell-cell adhesions and thus protect cells in the outer layer of biofilms from ethanol, a function primarily dependent on cell-cell adhesions. This work offers a possible explanation for how biofilm formation is regulated during the immobilized fermentation process, and can enhance environmental tolerance in industrial production.

## Introduction

Similar to human cities, biofilms are groups of microorganisms in which cells collaborate together and produce matrices of extracellular polymeric substance to survive (Kuhn et al., 2002). Biofilms protect cells from various external stimuli such as osmostress, heat shock, oxidative stress, and nutrient deficiencies (Blankenship and Mitchell, 2006). The difficulty of removing biofilms formed on medical devices and catheters *in vivo* by pathogenic microorganisms such as *Pseudomonas aeruginosa, Candida albicans*, and *Staphylococcus aureus* using antibiotic treatments presents a significant problem in medicine (Almirante et al., 2006). These characteristics, however, are beneficial in biofilm reactors. These reactors are formed by immobilized cells adhering to solid surfaces, and demonstrate excellent tolerance against substrates/products inhibition as well as higher reuse efficiency for immobilized strains in batch fermentation (Pal and Khanum, 2011). Repeated batch fermentations in biofilm reactors have been applied to efficiently produce ethanol fuel using *S. cerevisiae* with a high optimal conversion rate (Li et al., 2012). During the ethanol fermentation process, biofilms have returned higher ethanol yields and shorter fermentation times were observed relative to the planktonic cell fermentation process (Germec et al., 2015).

Biofilm formation occurs primarily through three phases: attachment, maturation, and dispersion. In response to certain environments, cells will attach to surfaces and begin producing exopolymeric substances (EPS, including polysaccharides, proteins, lipids and nucleic acids) to form a complex three-dimensional architecture (Suresh Kumar et al., 2007). Although the specific compositions of EPS remain unknown, EPS and especially their proteins have been found to support biofilm structures (Fong and Yildiz, 2015) and perform biochemical activities, such as the protease inhibitor found in *Pseudomonas aeruginosa* biofilm EPS that protects cells from proteolytic attacks (Tseng et al., 2018).

Adhesion genes, which contribute to the ability of cells to adhere to other cells or surfaces, are widely studied in biofilm formation (Stringer and Keely, 2001; Bojsen et al., 2012). S. cerevisiae carries a family of adhesive special surface glycoproteins encoded by FLO1, FLO5, FLO9, FLO10, and FLO11 (Teunissen and Steensma, 1995). They share similar but slightly different structures, and serve different functions (Halme et al., 2004). FLO1, FLO5, FLO9, and FLO10 confer cell-cell adherence and contribute to flocculation (Guo et al., 2000). FLO11 is responsible for cell-surface adhesions and required for agar invasive growth, pseudohyphae, and biofilm formation (Reynolds and Fink, 2001; Zara et al., 2005; Verstrepen and Klis, 2006); however, numerous types of these adhesions are present in different yeast species (Douglas et al., 2007). FLO11 is primarily recognized as the only FLO gene to confer surface adhesion and is thus required for several important developmental transitions including adherence to agar and plastic surfaces in many yeast strains, especially the widely studied S. cerevisiae Σ1278b (Guo et al., 2000; Reynolds and Fink, 2001). Differing from Σ1278b, the S288c strain requires FLO1 for biofilm formation as well as FLO11, and their regulations differ (Fichtner et al., 2007). In S. cerevisiae wine strains, FLO5 has been proven to drive adhesive properties which depend on surface adhesion ability (Di Gianvito et al., 2017). Several cell surface proteins besides FLO genes participate in yeast biofilm formation. Glycosylated cell surface proteins, encoded by CCW14 and YGP1, contribute to yeast biofilms. This may result from the hydrophobicity of the two proteins in haploid strains (Moreno-García et al., 2018).

Some biofilm findings have been based on cells exposed to stress conditions such as low pH and glucose (Reynolds and Fink, 2001; D’Urzo et al., 2014), heat shock (Grudniak et al., 2015), or oxidative stress and osmostress (Geier et al., 2008; Watanabe et al., 2013). By screening deletion mutants and overexpression strains (Andersen et al., 2014), a number of regulators have been shown to control yeast biofilm formation including MAPK (Madhani and Fink, 1997; Gagiano et al., 2003; Chavel et al., 2014), PKA (Villa et al., 2017), and main glucose repression (Lambrechts et al., 1996; Bester et al., 2012; Nakagawa et al., 2017) pathways. To understand the biofilm formation mechanisms observed in biofilm reactors during fermentation, several studies have employed biofilm and free cell comparisons (Li et al., 2015; Liu et al., 2016). These studies have uncovered the involvement of carbohydrates, amino acids, signal transduction, and oxidoreductase metabolism in biofilm formation. In the industry strain *S. cerevisiae* 1308 used in this laboratory, glycolysis and gluconeogenesis metabolism have been found to play key roles in the development of *S. cerevisiae* biofilms by comparing the transcriptomes of biofilms and free cells (Li et al., 2015).

*MIG1*, which encodes a C2H2 zinc finger protein, inhibits *GAL* gene expression in the presence of glucose and has been recognized as a main effector in the glucose repression pathway (Cao et al., 2011; Liu et al., 2011; Li et al., 2015). *NRG1*, which represses *FLO11* by binding to its promoter, shares similar functions with *MIG1* in the glucose repression pathway (Kuchin et al., 2002). When glucose concentrations drop, the activated *SNF1* kinase complex can phosphorylate and inactivate repressors *MIG1* and *NRG1* (Bendrioua et al., 2014). *FLO11* is supposed to be repressed by *MIG1* as well as *NRG1* in glucose repression (Gancedo, 1998; Winderickx et al., 2003). Conversely, an overexpression of *MIG1* induces filamentous growth – a morphology confirmed to be primarily under the control of *FLO* genes (Karunanithi and Cullen, 2012). The effect of *MIG1* on the expression of *FLO11* and biofilm formation, however, has remained indeterminate in past research.

To explore the effect of transcription regulator *MIG1* in yeast biofilm formation, the present study utilized transcription to analyze *MIG1* and *FLO* genes expression changes in industrial yeast during biofilm formation, verifying the effects of these genes on biofilm formation. It was found that in addition to *FLO11*, three other *FLO* genes (*FLO1, FLO5*, and *FLO9*) are essential for *S. cerevisiae* biofilm formation during immobilization. Furthermore, *MIG1* may function as a regulator of *FLO1, 5* and *9* genes, as they responded to *MIG1* expression changes during biofilm formation.

## Materials and Methods

### Yeast Strains and Growth Conditions

*Saccharomyces cerevisiae* 1308 (Chen et al., 2013) is a diploid industrial strain isolated from fermentative habitats and commonly grown in solid yeast extract peptone dextrose medium (1% yeast extract, 2% peptone, 2% glucose and 2% agar) at 30°C. In this study, yeast strain cultures were grown in liquid yeast extract peptone dextrose medium (1% yeast extract, 2% peptone, and 2% glucose). Fermentation experiments were performed in a fermentation medium containing 20% glucose, 0.4% peptone, 0.4% (NH_4_)_2_SO_4_, 0.3% yeast extract, 0.3% KH_2_PO_4_, 0.05% MgSO_4_, 0.005% ZnSO_4_⋅7H_2_O, and 0.005% FeSO_4_⋅7H_2_O. To select yeast transformants, G418 Sulfate (345180, Merck, Japan) was added at final concentrations of 400 and 800 μg/mL to solid yeast extract peptone dextrose medium (YPD).

Ethanol fermentations were performed by adding 1 mL overnight cultures to 250 mL flasks already containing 100 mL fermentation medium in the presence or absence of 4 g dry cotton fiber. Flasks were placed on a shaker activated at 250 rpm/min and maintained at 35°C. Continuous batch fermentation was conducted for the immobilized culture; in these, “waste broth” was removed and fresh broth was added as residual glucose was depleted (<1 g/L).

### RNA Preparation, cDNA Library Construction and Transcription Profiling Data Analysis

Biofilm cells were isolated from cotton fibers via ultrasonication at three different stages during biofilm development. Planktonic and biofilm cells were collected and washed twice in PBS. Cell pellets were immediately frozen in liquid nitrogen and stored at -80°C. Three biological replicates were prepared from the samples taken under each condition. RNA was isolated from both free and biofilm *S. cerevisiae* cells using the methods previously described (Cao et al., 2006). A cDNA library was constructed using published methods (Li et al., 2015). The reads per kilobase transcriptome per million mapped reads method (RPKM; Mortazavi et al., 2008) was applied to calculate the expression levels of selected genes. Furthermore, the works of Audic and Claverie (1997) were used to determine the significance of the digital gene expression profiles. This study selected a level of FDR ≤0.001 and absolute value of Log_2_Ratio ≥1 as criteria for assessing the significance of differential gene expression.

### Construction of Deletion and Complemented Mutants

*Saccharomyces cerevisiae* strain mutants were constructed by deleting corresponding genes in *S. cerevisiae* 1308, the selected industrial yeast strain, using the homologous recombination system (LFH-PCR: PCR-Synthesis of disruption cassettes with long flanking homology) according to published methodology (Nikawa and Kawabata, 1998). Knock-in component construction is described below. The PCR primers used in this study are listed in **Table 1**. PCR-generated DNA molecules (knock-in components) consisted of a KanMX marker cassette, as KanMX sequences show G418 resistance in *S. cerevisiae* and Kanamycin resistance in *Escherichia coli*. KanMX marker cassettes with long homologous regions (450–500 bp) flanking the target locus was then used for directed gene alterations in *S. cerevisiae*. Knock-in components were then transformed into competent *S. cerevisiae* 1308 cells using a Bio-Rad electroporation systems set at 1.5 kV, 25 mF with a 200 Ohm pulse controller. The sorbitol transformation method was used.

**Table 1:** The sequences of the oligonucleotide primers used in this study.

Primer name	Primer sequence	Source
*MIG1*-up-F	ACTTGTTCGAGCTCTTGAGTTCTCCTGGC	This work
*MIG1*-up-R	AGGAGGGTATTCTGGGCCTCCATGTCGCCTCTGACTTCGCAGCTACTTTGGACTT	This work
*MIG1*-dn-F	ATCGTATGTGAATGCTGGTCGCTATACTGCGAGGTAAAAGAGGCAGAAAGAAGAAGGT	This work
*MIG1*-dn-R	ATAACAGTGTTGGAATAACGTGGTGAAAG	This work
G418-*MIG1*-F	TCCAAAGTAGCTGCGAAGTCAGAGGCGACATGGAGGCCCAGAATACCCTCCTTGACAGT	This work
G418-*MIG1*-R	TCTTCTTTCTGCCTCTTTTACCTCGCAGTATAGCGACCACCAGCATACGATTGACG	This work
*FLO11*-up-F	AGGGTACGATTGTTTCTAGAGAAATGTG	This work
*FLO11*-up-R	GTCGACCTGCAGCGTACGAGTGTGCGTATATGGATTTTTGAGGCCTAC	This work
*FLO11*-dn-F	CAGATCCACTAGTGGCCTATGCGTGATACAATTCCAACATGTTCGTTTC	This work
*FLO11*-dn-R	GATTATTAGTTGTGCCAAGGCAATATC	This work
G418- *FLO11*-F	TCCATATACGCACACTCGTACGCTGCAGGTCGACAACCCT	This work
G418-*FLO11*-R	CATGTTGGAATTGTATCACGCATAGGCCACTAGTGGATCTGATATCAC	This work
*FLO1*-up-F	TTCTTCTCCAGTCATTTCTTCCTCAGTCATTTCTTCTTCTAC	This work
*FLO1*-up-R	GGTATTCTGGGCCTCCATGTCCTACCGTGGTTTGTTTT	This work
*FLO1*-dn-F	TGCTGGTCGCTATACTGCCTGCCATTGTTTCGAC	This work
*FLO1*-dn-R	GCAATAAGGACGCAATGAAGACACTTAAACCACTACCGG	This work
G418- *FLO1*-F	AAAACAAACCACGGTAGGACATGGAGGCCCAGAATACC	This work
G418-*FLO1*-R	GTCGAAACAATGGCAGGCAGTATAGCGACCAGCA	This work
*FLO5*-up-F	TTATTGTCATCAGAACTCCAACTACTGCCATCTCATCCAGTT	This work
*FLO5*-up-R	TATTCTGGGCCTCCATGTCGCAGGATGTCACGGTAA	This work
*FLO5*-dn-F	ATGCTGGTCGCTATACTGTACAATTTCTTCTTGTGAATCTGACA	This work
*FLO5*-dn-R	TGCTCAACCCGGAACTTGTTAGACTCATGGTGTT	This work
G418- *FLO5*-F	TTACCGTGACATCCTGCGACATGGAGGCCCAGAATA	This work
G418-*FLO5*-R	TGTCAGATTCACAAGAAGAAATTGTACAGTATAGCGACCAGCAT	This work
*FLO9*-up-F	TAAAACTAGTTTAAGTTTCTGGCGACCCTCCTGGAATGCTTACCTT	This work
*FLO9*-up-R	TTCTGGGCCTCCATGTCTTTTGGGGCTTTTATTGT	This work
*FLO9*-dn-F	GGTCGCTATACTGCAAAGGAATTGGTGCTTGTTCTAATCCAATA	This work
*FLO9*-dn-R	GTATAATTTGAAGGTCTGGAATGGTACAGTTTGGCTGGCT	This work
G418- *FLO9*-F	ACAATAAAAGCCCCAAAAGACATGGAGGCCCAGAATACC	This work
G418-*FLO9*-R	GCACCAATTCCTTTGCAGTATAGCGACCAGCATTCACAT	This work
pAurR-*MIG1*-F	CTGGTACCCGGGTCGACATGCAAAGCCCATATCCAAT	This work
pAurR-*MIG1*-R	TAGTTAACCTCTAGATCAGTCCATGTGTGGGAAGG	This work
pAurR-*FLO11*-F	CTGGTACCCGGGTCGACATGCAAAGACCATTTCTACT	This work
pAurR-*FLO11*-R	AGGTCAACATAAGATTTCAGTCCATGTGTGGGAAGG	This work

Complemented strain *MIG1*Δ + *pMIG1* and *FLO11*Δ + *pFLO11* were constructed by expressing *MIG1* and *FLO11* in strain *MIG1*Δ and *FLO11*Δ by plasmid pYX212-AurR respectively. Plasmids were constructed using ClonExpress^®^ One Step Cloning Kit. *MIG1* and *FLO11* were amplified with primers pAurR-*MIG1*-F(R) and pAurR-*FLO11*-F(R) from *S. cerevisiae* genome, then it were gel purified and ligated to lineated linearized plasmid pYX212-AurR (using restriction enzyme XbaI and SalI) Plasmid were first constructed in E. coli DH-5α, and then transformed into S. cerevisiae 1308 mutants respectively. All the primers and sequence information can be found as **Table 1**.

### qRT-PCR Analysis

RNA extractions and quality control experiments were performed as described in the previous section. Reverse transcription was performed using an AMV First Strand cDNA Synthesis Kit (Sangon Biotech) according to standard protocols. Primer 5 software was used to select the primers. The analyzed genes and primers used for analysis are listed in **Table 2**. Quantitative real-time PCR (qRT-PCR) assays were performed using SYBR Green PCR Master Mix (Applied Biosystems) in a StepOnePlus Real-Time PCR System. Reactions were performed according to manufacturer instructions, and three technical replicates with one negative control were performed for each sample. Gene transcription levels were determined according to the 2^−ΔΔCT^ method, using 18s rRNA and *FBA1* as reference genes for normalizing gene expression levels (Tofalo et al., 2014; Li et al., 2015; Nadai et al., 2015).

**Table 2:** Genes and primers for quantitative real-time PCR.

Gene	5′-3′ forward primer sequence	5′-3′ reverse primer sequence
*FLO1*	GCGTTCAACTGTTGT GCTCAA	GATACCGTCAATGGTAAA GTTCGTT
*FLO5*	TTGGCCTTTCTGGCA CTAATTAA	TCCTCTGGCCTGCTG GTAAG
*FLO9*	GGGTTCTTACACATTC AAGTTTGCT	GCAATGCTACCAC CGACTGA
*FLO1*	AATCACATAGAACATCG CCCACTA	TCGATTTCAACGCCT GAAGA
*FLO11*	ACTTTGGATGTGACTT CCGTTTC	ACCTTTGACATGAATAGTG ATTTGGTA
*MIG1*	TCTCCCAAAACGATGGCTAA	ACTATGGCTATTGCT CAACGAA
*18S*	ACGGAGCCAGCGAGT CTAAC	CGACGGAGTTTCACAAG ATTACC
*FBA1*	GCTTACGGTATCCCAGTTG TCTTAC	CGAACCATGGCAA CAACTTCT

### Biofilm Formation on Plastics

Yeast strains were grown in YPD overnight at 30°C. After collection and washing, cells were resuspended in YPD at an OD600 of 1 and transferred to the wells of a microtiter plate where they were incubated for 24 h at 30°C. Four replicate wells were used for each treatment. Biofilm-containing wells were washed twice in 200 μL PBS to remove free cells. Biofilms were then stained with 1% crystal violet, after which wells were washed repeatedly with water and photographed. For quantification, crystal violet was solubilized by adding 100 ml of acetic acid, plates were incubated for 15 min, and the absorbance at 570 nm was measured using a microplate reader.

### Confocal Laser Scanning Microscopy (CLSM) Analysis

Biofilm cells growing on cotton fiber media were harvested and immediately stained with FUN-1 and Alexa Fluor 488-conjugated ConA (both from Molecular Probes, Inc., Eugene, OR). FUN-1 (excitation wavelength: 543 nm; emission wavelength: 560 nm) is converted to an orange-red molecule in metabolically active cells, while Alexa-Fluor 488-conjugated ConA (excitation wavelength: 488 nm; emission wavelength: 505 nm) binds to the glucose and mannose residues of cell-wall polysaccharides, displaying green fluorescence. Confocal images were captured using a Leica TCS SP5 II.

### Flocculation Assay

Strains were grown in YPD medium for 12 h at 30°C. After being diluted to equivalent OD600 levels, each strain was placed in a separate test tube. The tubes were then vibrated thoroughly to suspend all contained cells and subsequently left to stand until all cells underwent sedimentation. Images were recorded at 5 min intervals, and flocculation ability was measured as the time required for sedimentation to complete.

### Standard Plate-Wash Assay

Cells were grown on standard YPD agar plates for 3 days. Observations indicated all strains grew equally well in this environment. Next, each plate was added to 1 ml water and shaken at 50 rpm for 2 min. The water was then discarded and images of the colonies were recorded.

### Fermentation Ethanol Resistance Test

First, yeast cells underwent a 3-day immobilization process on cotton fiber under the previously described immobilization conditions (see section ‘Planktonic cultivations and biofilm fermentation’). When the ethanol resistance test was started, the residual medium was discarded into flasks and fresh medium containing selected glucose concentrations was added (other ingredients were the same as above). Different ethanol volumes, ranging from 5% (v/v) to 15% (v/v), were added and 1 ml of samples were drawn from each flask at 4 h intervals. Samples underwent the following procedures: first, samples were centrifuged at 10,000 rpm for 1 min to sediment cells; next, the supernatant was transferred to another 1.5 ml centrifuge tube; and finally, the glucose concentration of the supernatant was tested using the DNS method (3, 5-Dinitrosalicylic acid).

### Statistical Analysis

All experiments were done in at least triplicate. The data presented are the means of three or more experiments. Significant differences were determined by Student’s *t*-test (*p* < 0.05).

## Results and Discussion

### Transcriptional Changes in *FLO* and *MIG1* Genes

**FIGURE 1:**
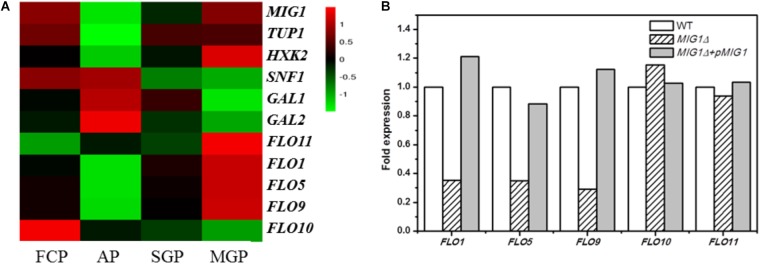
Transcriptional proofing and qRT-PCR results. **(A)** Transcriptional levels of *MIG1*, its regulated genes and *FLO* genes in FCP (free cells period), biofilm cells at AP (attachment period), SGP (sessile growth period), and BMP (biofilm maturation period). **(B)** qRT-PCR results. Relative expression of *FLO* genes in *MIG1*Δ and *MIG1*Δ + *pMIG1* compared with wild-type respectively.

Whole-genome expression profiling was performed to explore the gene expression differences between biofilm-forming cells and planktonic cells, comparing gene expression during the biofilm attachment (3 h), sessile growth (14 h), and biofilm maturation (30 h) periods (based on mRNA RPKM values). Genes whose expression change by over 2.0 fold were recognized as significantly regulated. Such RNA-Seq results have been proven accurate by Li et al. (2015). Comparing biofilm and planktonic cells, the *FLO11* gene was up-regulated by 6.8-fold, 5.0-fold, and 18.4-fold in the three selected periods. *FLO1, 5*, and *9* were down-regulated at the attachment period, but up-regulated during the maturation period (biofilm vs. planktonic). The expression levels of these *FLO* genes increased during the biofilm formation process, especially in the maturation period. A different flocculation gene, *FLO10*, was down-regulated in all three periods (biofilm vs. planktonic), with its expression level in biofilm cells decreasing during biofilm formation (**Figure 1A**).

Among the investigated genes, the transcription factor gene *MIG1* attracted attention with a significant, 27-fold down-regulation during the attachment period, whereas the *FLO11* gene, hypothetically located downstream, was up-regulated throughout biofilm formation (biofilm vs. planktonic, as noted above). When biofilm cells were compared across the three periods, however, the expression levels of *MIG1* increased alongside *FLO11* (**Figure 1A**). When compared with biofilm cells at attachment, *MIG1* was up-regulated 14- and 26-fold during the sessile growth and maturation periods, respectively. *SNF1*, the encoded primary subunit of the SNF1 kinase complex which can inactivate and repress *MIG1* (Bendrioua et al., 2014), was down-regulated. This was especially true during the maturation period when *MIG1* expression was maximized in biofilm cells, supporting the theory that *MIG1* repression became stronger in later periods. *GAL1* and *GAL2*, which are repressed by *MIG1* (Cao et al., 2011), were not significantly down-regulated in biofilm cells during the maturation period relative to the attachment period (**Figure 1A**). Furthermore, the expression of *HXK2*, also repressed by *MIG1* (Fichtner et al., 2007; Peláez et al., 2012), increased alongside *MIG1* during biofilm formation (**Figure 1A**).

The down-regulated expression of *FLO10* was consistent with the results of Verstrepen and Klis (2006), who found that *FLO10* expression conferred weak flocculation. The up-regulation of *FLO1, 5*, and *9* during later periods suggests that cell-cell adhesions play a role in biofilm fermentation. In biofilm cells, the expression levels of *FLO1, 5*, *9*, and 11 increased alongside *MIG1* in all three periods of biofilm formation. The varying trends in *FLO11* transcription levels resembled those of *MIG1*, contrary to previous reports that *MIG1* represses *FLO11* (Verstrepen and Klis, 2006). *MIG1* can function as a transcriptional activator in some contexts, particularly in cells lacking the chromatin-remodeling protein encoded by *TUP1* (Treitel and Carlson, 1995) which was down-regulated in the biofilm cells investigated here (**Figure 1A**). Additionally, the *GAL1, GAL2*, and *HXK2* genes repressed by *MIG1* were not down-regulated during *MIG1* overexpression in the later periods of biofilm formation. These observations failed to illustrate the transcription repressor actions of *MIG1* during biofilm formation. In addition, overexpressed *MIG1* induced filamentous growth which morphology mainly controlled by *FLO* genes (Karunanithi and Cullen, 2012). Further studies will thus be required to uncover the role of *MIG1* in *FLO11* expression and yeast biofilm formation.

### *MIG1* and *FLO* Genes Function in Biofilm Formation

**FIGURE 2:**
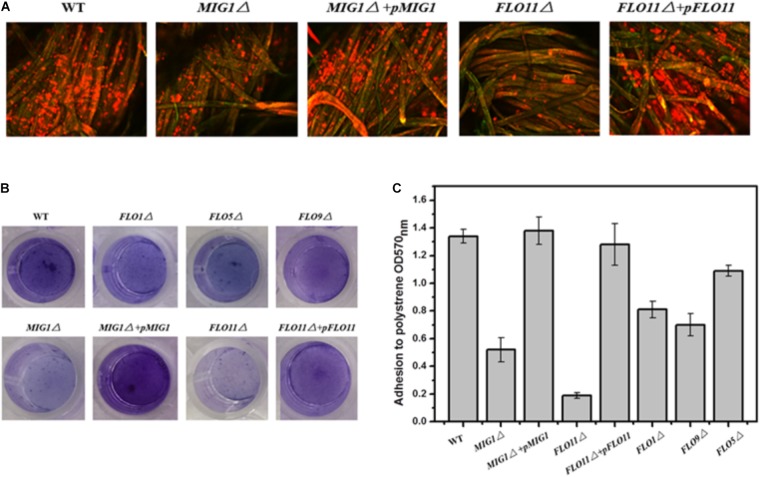
Biofilm formation. **(A)** CLSM images of wide-type, *MIG1*Δ, *MIG1*Δ + *pMIG1*, *FLO11*Δ and *FLO11*Δ + *pFLO11* biofilms formed on fibers after 24 h fermentation. Strains were dyed orange-red by FUN-1 and polysaccharides were dyed green by Alexa Fluor 488-conjugated ConA. **(B)** Adherence abilities of wide-type, *FLO1*Δ, *FLO5*Δ, *FLO9*Δ, *MIG1*Δ, *MIG1*Δ + *pMIG1*, *FLO11*Δ, and *FLO11*Δ + *pFLO11* strains were assayed after incubation in polystyrene 24 h. Wells were washed twice with PBS (in 200 μL) to move away free cells and stained with 1% crystal violet. Wells were washed repeatedly with water and photographed. **(C)** Adhesion was expressed as OD570 and was measured by solubilizing crystal violet in acetic acid.

To investigate the role of *MIG1* in yeast biofilm formation, *MIG1* deletion and complemented strains were generated from the wild-type strain. To verify the effects of flocculation genes on the ability of the selected industry strain to form biofilms, mutant strains *FLO1*Δ, *FLO5*Δ, *FLO9*Δ, and *FLO11*Δ were constructed. Confocal laser scanning microscopy (CLSM) was utilized to observe the biofilms formed on fibers, which live in self-produced matrices of hydrated EPS, primarily polysaccharides (Flemming and Wingender, 2010). FUN-1 and Alexa Fluor 488-conjugated ConA were used to dye live cells red-orange and polysaccharides green respectively. Biofilms were formed after 72 h on cotton fiber substrates. Attachments to the immobilization carrier were less obvious in *MIG1*Δ and *FLO11*Δ strains relative to the wild-type. It also can be observed that biofilms formed by *MIG1*Δ + *pMIG1* and *FLO11*Δ + *pFLO11* were restored (**Figure 2A**). As the attachments of *FLO1*Δ, *FLO5*Δ, and *FLO9*Δ on the immobilization carrier appeared identical to those of the wild-type, their data are not shown.

The photographs recorded via CLSM were used to roughly evaluate the biofilms formed by these strains. To estimate and distinguish the biofilms of each *S. cerevisiae* strain, biofilm formation during growth in 96-well plates was quantified using the crystal violet staining method. All deletion mutant strains demonstrated decreases in biofilm formation compared to the wild-type (**Figure 2B**). *FLO11*Δ formed only 1/7 of the biofilm produced by the wild-type, the least among these mutant strains. The mutant strains in order decreasing biofilm formation were *FLO9*Δ > *FLO1*Δ > *FLO5*Δ > *MIG1*Δ > *FLO11*Δ. Two complemented strains *MIG1*Δ *+ pMIG1* and *FLO11*Δ + *pFLO11* formed biofilms as much as wild-type (**Figure 2C**).

*FLO11* has been widely recognized as important in cell-substrate interactions and biofilm formation in yeast. Other *FLO* genes influence cell–cell interactions and exert an invisible effect on biofilm formation (Guo et al., 2000; Halme et al., 2004). The reduction in biofilm formation in both *FLO11*Δ in these tests and Σ1278b confirms that *FLO11* plays an important role in yeast biofilm formation. However, *FLO1* was also required for biofilms in the S288c strain, and its regulation differed from *FLO11* (Fichtner et al., 2007). In the 1308 strain used in this study, *FLO1, 5*, and *9* were all required for biofilm formation. This differed from Σ1278b and S288c (Douglas et al., 2007; Van Mulders et al., 2009). These observations indicated that disabling *FLO1, 5, 9*, and *11* genes, as well as *MIG1*, vitiates biofilm formation ability. Cell-cell and cell-surface adhesion abilities were both required for yeast biofilm formation.

### Expression Levels of *FLO* Genes in *MIG1* Deletion and Complemented Strain Biofilm Cells

As the transcription repression activities of *MIG1* were significantly down-regulated during the biofilm attachment period (biofilm vs. planktonic), the decrease in biofilm formation observed in *MIG1*Δ was unexpected. qRT-PCR tests were performed on both the wild-type, *MIG1*Δ and *MIG1*Δ + *pMIG1* cells to examine the expression of *FLO* genes in biofilm-forming cells (**Figure 1B**). Expressions in the wild-type condition were defined as 100%, and expressions in mutant strains were calculated as relative numbers to evaluate the effects *MIG1* during biofilm formation. The *FLO1, 5*, and *9* genes were down-regulated in *MIG1*Δ; however, no pronounced expression difference were observed in the *FLO10* and *FLO11* genes when 18s rRNA was used as the reference gene. This result was supported when *FBA1* was used as an alternative reference gene (**Supplementary Figure S1**). Expression levels of these genes in *MIG1* complemented strain were increased compared with genes in deletion mutant.

According to the qRT-PCR results, *FLO1, 5*, and *9* were down-regulated in *MIG1*Δ as compared to the wild-type. Expression levels of these genes were restored to original level in *MIG1* complemented strain. This same expression pattern between *MIG1* and the *FLO1, 5*, and *9* genes was observed in biofilm formation, suggesting these *FLO* genes were regulated by *MIG1*. Mature biofilms were divided into three parts: the outer, intermediate, and inner surface layers (Jiang et al., 2015). Although cells-surface adhesion is a primary step in biofilm formation and critical for forming the inner biofilm layer (Periasamy et al., 2012), cell–cell adhesions appeared more requisite in the intermediate and outer layers. The expressions of *MIG1* and *FLO1, 5*, and *9* genes decreased during the attachment period but became over-expressed in the maturation period, indicating adhesions are required for biofilm formation. *FLO11* was neither up- nor down-regulated in *MIG1*Δ or *MIG1*Δ + *pMIG1* relative to the wild-type, indicating *FLO11* was not regulated by transcription factor *MIG1* in the yeast biofilm formation process.

### *MIG1* Regulated Yeast Flocculation Ability and Invasive Growth

The regulation *MIG1* exerted on *FLO1, 5*, and *9* gene transcriptions indicated it may affect the yeast flocculation ability and adhesion-dependent invasive growth. Phenotype changes were observed between the mutant strains.

**FIGURE 3:**
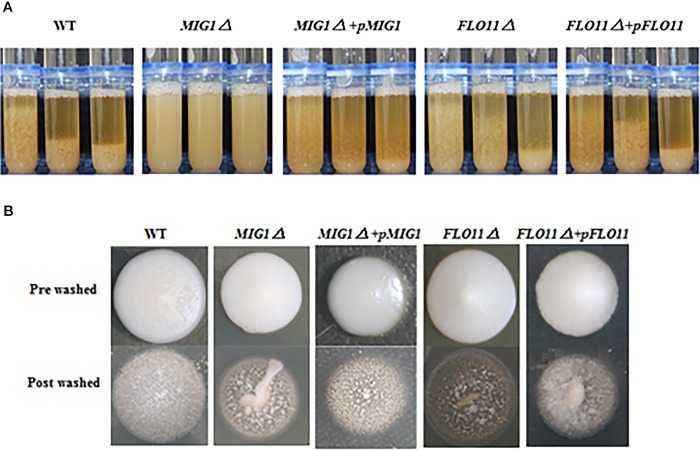
Invasive growth and flocculation ability. **(A)** Wild-type, *MIG1*Δ, *MIG1*Δ *+ pMIG1*, *FLO11*Δ, and *FLO11*Δ *+ pFLO11* strains were photographed every 5 min. **(B)** Plate wash tests of wide-type, *MIG1*Δ, *MIG1*Δ *+ pMIG1*, *FLO11*Δ, and *FLO11*Δ *+ pFLO11*. Strains were grown on YPD agars for 3 days, same amount of water were added and plates were placed on the shaker for 10 min. Photos of pre-washed strains and post-washed strains were taken.

To illustrate the effect of *MIG1* on flocculation ability, the *MIG1*and *FLO11* mutant and complemented strains were compared (**Figure 3A**). The *MIG1*Δ strain lost a majority of its flocculation ability and *MIG1*Δ + *pMIG1* restored the ability; conversely, *FLO11* deletion and complemented had no influence on flocculation. A plate-wash test was performed to evaluate the impact of *MIG1* on invasive cell growth (**Figure 3B**). In this test, *FLO11*Δ was observed to have the fewest colonies remaining on the agar plate, suggesting a weakened invasive ability. *MIG1*Δ also demonstrated slightly decreased invasive growth. The invasive growth was restored by overexpressing the two genes in its corresponding mutant strains respectively.

The loss of flocculation ability and decreased expression of the *FLO1, 5*, and *9* genes in *MIG1*Δ confirmed that *MIG1* play an important role in cell–cell adhesion by regulating the expression levels of those genes. *FLO11* was not required for cell–cell adhesion, as reported by Fichtner et al. (2007). Less obviously, the decrease in invasive growth shown by *MIG1*Δ may have been caused by a reduction in the invasive growth glycoproteins encoded by *FLO1, 5*, and *9* which regulated biofilm formation with two possible aspects: first, *FLO1, 5*, and *9*-dependent cell–cell adhesions were required for the intermediate and outer layers of mature biofilm, and these genes were overexpressed during appropriate formation periods. Second, the expression levels of *FLO1, 5*, and *9* influenced cell-surface adhesion, suggesting these *FLO* genes influence the maintenance of stability in inner layers.

### *MIG1* Function on Ethanol Resistance

In fermentation experiments, conducted to investigate the abilities of mutant strain biofilms to survive during the accumulation of high ethanol concentrations, strain growth was inhibited and glucose consumption rates decreased when ethanol concentrations were high. The residual glucose concentrations were measured to demonstrate the environmental resistance ability of each strain.

**FIGURE 4:**
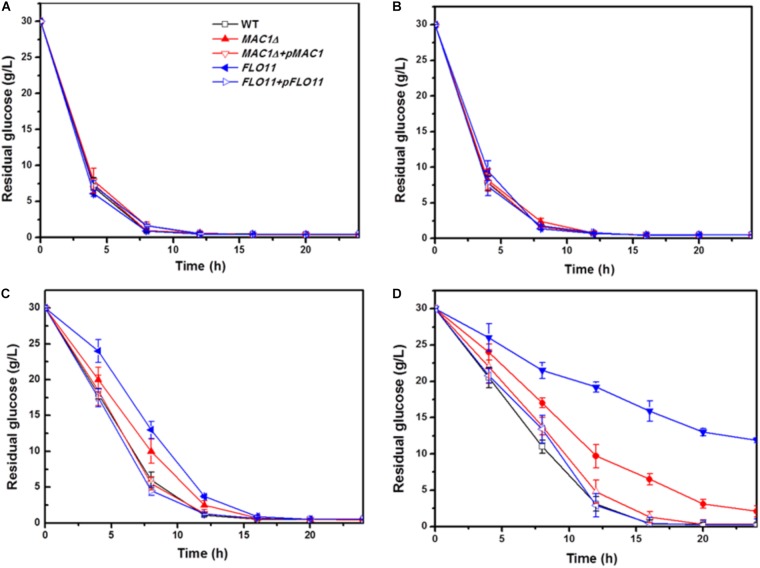
Glucose concentration change during fermentation in different ethanol concentration. **(A–D)** Residual concentrations of glucose were measured under 0, 5, 10, and 15% ethanol concentrations, in biofilm state fermentation.

In the 0 and 5% ethanol groups, glucose was depleted within 8 h. Although the glucose consumption rates of these strains in 10% were slower than in 5% group, there was no obvious difference among the three strains in a same group (**Figures 4A,B**); however, the consumption rates of all strains were slower in 10% ethanol relative to 5%. In the 10% ethanol group, both wild-type and *MIG1*Δ strains depleted their glucose supply at approximately 12 h, whereas *FLO11*Δ took 16 h. The glucose consumption rates of *MIG1*Δ and *FLO11*Δ were both slower than that of the wild-type (**Figure 4C**). When ethanol concentrations reached 15%, the wild-type strain had depleted all available glucose after 16 h. At 24 h, however, there were 2 g/L and 12 g/L glucose remaining in the *MIG1*Δ and *FLO11*Δ samples, respectively (**Figure 4D**). *MIG1* and *FLO11* complemented strains depleted glucose faster than its deletion strains in 10 and 15% ethanol groups.

Previous studies have shown that *FLO11* contributes to biofilm formation, and is therefore required for environmental resistance (Váchová et al., 2011). The present results confirmed that *FLO11* deficiency abated the ethanol tolerance of cell strains in biofilm fermentation. Flocculation, dependent on cell–cell adhesion, plays an important role in the ethanol resistance of cells as well as biofilm formation in many yeast strains. Flocculation has been restored by an overexpression of *FLO11* in laboratory strain S288C, protecting inner cells from multiple stresses including high ethanol concentrations (Smukalla et al., 2008). Furthermore, flocculation strains showed high levels of *FLO5* expression and significant resistance to ethanol stress (Tofalo et al., 2014). Cell–cell adhesions, which occur during the later biofilm formation stages, play important roles in ethanol resistance and cell-surface adhesion. The decreased ethanol resistance of *MIG*1**Δ may have resulted from decreased cell–cell and cell–surface adhesions. *FLO11*Δ showed weaker biofilm formation and ethanol resistance than *MIG1*Δ, suggesting cell–surface adhesions are a primary factor in ethanol resistance.

## Conclusion

This study focused on elucidating the roles played by the *MIG1* (transcription factor) and *FLO* (adhesion) genes in yeast biofilm formation and ethanol resistance. Results showed *FLO11*, which confers cell-surface adhesion abilities, played an important role in yeast biofilm formation but was not repressed by transcription factor *MIG1. MIG1* regulating the expression levels of *FLO1, 5* and *9* which affected yeast biofilm formation respectively. According to these findings, this work presents the possibility that *FLO1, 5*, and *9* gene expressions were increased at later stages and high ethanol concentrations, regulated by *MIG1*, in order to protect cells by keeping or increasing the outer layer of biofilms via enhanced cell-cell adhesions. The present work serves as a basis for future studies to examine the complex network systems that regulate *S. cerevisiae* biofilm formation and maintenance, as more work will be necessary to elucidate the regulation pathways by which *MIG1* influences these *FLO* genes in *S. cerevisiae* biofilm development.

## Availability of Data and Material

The datasets supporting the conclusions of this article are included within the article and its additional files.

## Author Contributions

LY participated in the design of the study, constructed the strains, participated in the fermentation experiments, drafted the manuscript, and revised the manuscript. CZ participated in the design of the study, the fermentation experiments, and drafted the manuscript. YC participated in the design of the study. HY conceived the study and participated in its design. All authors have read and approved the final manuscript.

## Conflict of Interest Statement

The authors declare that the research was conducted in the absence of any commercial or financial relationships that could be construed as a potential conflict of interest.

## Supplementary Material

FIGURE S1 Relative expression of *FLO* genes in *MIG1*Δ compared with wild-type respectively using *FBA1* as reference gene.
