# Supplementary figures and images for: FLO Genes Family and Transcription Factor MIG1 Regulate Saccharomyces cerevisiae Biofilm Formation During Immobilized Fermentation
Source: Front Microbiol. 2018 Aug 23;9:1860. doi: 10.3389/fmicb.2018.01860 (PMC6119776; doi:10.3389/fmicb.2018.01860)

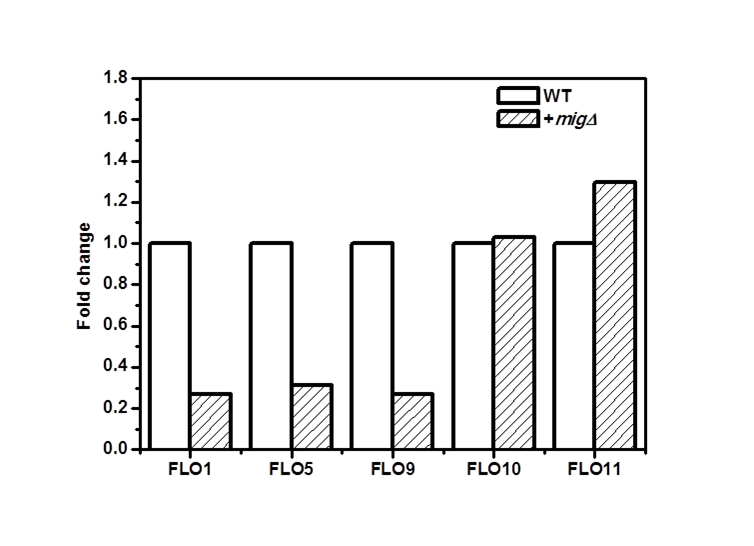

Supplement: FIGURE S1 — Relative expression of FLO genes in MIG1Δ compared with wild-type respectively using FBA1 as reference gene. [file Image_1.TIF]
